# Hemorheological profiles and chronic inflammation markers in transfusion-dependent and non-transfusion- dependent thalassemia

**DOI:** 10.3389/fmolb.2022.1108896

**Published:** 2023-01-09

**Authors:** Patrizia Caprari, Elisabetta Profumo, Sara Massimi, Brigitta Buttari, Rachele Riganò, Vincenza Regine, Marco Gabbianelli, Stefania Rossi, Roberta Risoluti, Stefano Materazzi, Giuseppina Gullifa, Laura Maffei, Francesco Sorrentino

**Affiliations:** ^1^ National Centre for the Control and Evaluation of Medicines, Istituto Superiore di Sanità, Rome, Italy; ^2^ Department of Cardiovascular and Endocrine-Metabolic Diseases and Aging, Istituto Superiore di Sanità, Rome, Italy; ^3^ Department of Infectious Disease, Istituto Superiore di Sanità, Rome, Italy; ^4^ Department of Oncology and Molecular Medicine, Istituto Superiore di Sanità, Rome, Italy; ^5^ Department of Chemistry, Sapienza University of Rome, Rome, Italy; ^6^ Thalassemia Unit, S. Eugenio Hospital, Rome, Italy

**Keywords:** thalassemia major, thalassemia intermedia, hemorheology, blood viscosity, endothelium, cytokines, chemokines, adhesion molecules

## Abstract

The rheological properties of blood play an important role in regulating blood flow in micro and macro circulation. In thalassemia syndromes red blood cells exhibit altered hemodynamic properties that facilitate microcirculatory diseases: increased aggregation and reduced deformability, as well as a marked increase in adherence to the vascular endothelial cells. A personalized approach to treating thalassemia patients (transfusions, iron chelation, and splenectomy), has increased patients’ life expectancy, however they generally present many complications and several studies have demonstrated the presence of high incidence of thromboembolic events. In this study the hemorheological profiles of thalassemia patients have been characterized to point out new indices of vascular impairment in thalassemia. Plasma viscosity, blood viscosities at low and high shear rates (η1 and η200, respectively), erythrocyte aggregation index (η1/η200), and the erythrocyte viscoelastic profile (elastic modulus G', and viscous modulus G") have been studied in transfusion-dependent and non-transfusion-dependent thalassemia patients. Moreover, the levels of inflammation biomarkers in thalassemia have been evaluated to investigate a relationship between the biomarkers, the disease severity and the rheological parameters. The biomarkers studied are the main components of the immune and endothelial systems or are related to vascular inflammation: cytokines (IL-2, IL-6, IL-10, IL-17A, TNF-alpha), chemokines (IL-8, MIP-1alpha), adipocytokines (leptin and adiponectin), growth factors (VEGF, angiopoietin-1), adhesion molecules (ICAM-1, VCAM-1, E-selectin, L-selectin), and a monocyte/macrophage activation marker (CD163). This study shows that transfusion-dependent thalassemia patients, both major and intermedia, have blood viscosities comparable to those of healthy subjects. Non-transfusion-dependent thalassemia intermedia patients show high blood viscosities at low shear rates (η1), corresponding to the flow conditions of the microcirculation, an increase in erythrocyte aggregation, and high values of the elastic G' and viscous G" modules that reflect a reduced erythrocyte deformability and an increase in blood viscosity. Levels of cytokines, chemokines and adhesion molecules are different in transfusion- and non-transfusion dependent patients and positive correlations between η1 or η1/η200 and the cytokines IL-6 and IL-10 have been observed. The evaluation of the hemorheological profiles in thalassemia can provide new indicators of vascular impairment and disease severity in thalassemia in order to prevent the onset of thromboembolic events.

## Introduction

Thalassemia is a genetic disorder of hemoglobin characterized by the absence or reduced globin chain synthesis. Ineffective erythropoiesis, hemolysis, anemia and iron overload are the keypoints of these syndromes. The excess of the unbound alpha-globin chains and their degradation products precipitate in red cell precursors giving rise to ineffective erythropoiesis in β-thalassemia, while the underproduction of α-globin chains of hemoglobin and the formation of β-4 tetramers (HbH) lead to premature hemolysis in α-thalassemia. Hemolysis and anemia stimulate the synthesis of erythropoietin, leading to an intense proliferation of the ineffective marrow, skeletal deformities and a variety of growth and metabolic abnormalities. The anemia is further exacerbated by splenomegaly, and the iron overload is determined by an increased iron absorption (Taher et al., 2018b; Marcon et al., 2018).

Three clinical and hematological conditions of increasing severity are recognized in relationship to the extent of unbalance between the α globin and β globin chains: thalassemia minor, that is, the carrier state generally clinically silent, thalassemia intermedia, and thalassemia major. Patients with β thalassemia major usually present with severe anemia in infancy and become transfusion dependent for life. The thalassemia intermedia is caused by thalassemia genotypes milder than the thalassemia major ones and/or co-inheritance of α and β thalassemia, the clinical severity ranges widely depending on the phenotypes and a wide variation in complications, from the not transfusion dependent patients who may suffer chronic anemia of variable severity, mainly in particular conditions such as infections and pregnancy, to the transfusion dependent patients. Also ageing increases the severity of chronic anemia.

Although the life expectancy of thalassemia patients has markedly improved, patients with thalassemia major and intermedia generally present many complications (Musallam et al., 2012; Taher et al., 2015; Pazgal et al., 2016). Some complications are common to thalassemia patients and are preventable by blood transfusions: extramedullary hematopoiesis, splenomegaly, leg ulcers, growth retardation, and skeletal abnormalities. Additional complications are more frequent in not transfusion dependent patients such as thrombosis, pulmonary hypertension, right heart failure, and gallstones infections (Taher et al., 2009; Taher et al., 2011; Cappellini et al., 2012; Taher et al., 2012; Pazgal et al., 2016; Saliba and Taher, 2016).

Several studies have demonstrated the presence of high incidence of thromboembolic events in thalassemia, more common in thalassemia intermedia than in regularly transfused thalassemia major. In these patients a chronic hypercoagulable state is evident (Taher, et al., 2006; Cappellini et al., 2010; Cappellini et al., 2012; Taher et al., 2018b; Marcon et al., 2018) partially due to the impairment of the natural endothelial anticoagulant system, where the trans-endothelial receptor thrombomodulin is increased and the coagulation inhibitors protein C and S are diminished.

Red blood cells (RBC) of thalassemia patients exhibit impaired flow properties that facilitate micro-circulatory disorders: enhanced aggregability, reduced deformability, as well as a marked elevation of adherence to endothelial cells. Levels of pro-coagulant micro-particles derived from endothelium, platelets, RBC and leukocytes are also elevated in thalassemia patients (Pérez et al., 2004; Pérez et al., 2006; Cappellini et al., 2010; Renoux et al., 2018). Elevated levels of adhesion proteins such as vascular cell adhesion molecule (VCAM)-1 in thalassemia patients (Oztürk et al., 2001; Moshtaghi-Kashanian et al., 2006; Butthep et al., 2015; El-Rasheidy et al., 2016; Gülhan et al., 2016) suggest that endothelial injury or activation may be a feature of thalassemia (Cappellini et al., 2010; Cappellini et al., 2012).

In addition increased levels of circulating activated endothelial cells and elevated tumor necrosis factor (TNF)-α, interleukin (IL)-1β and vascular endothelial growth factor (VEGF) have been observed in β-thalassemia patients, which play an important role in the recruitment of leukocytes and erythrocytes and promote thrombosis at vascular inflammation sites (Kyriakou et al., 2001; Shitrit et al., 2008; El-Rasheidy et al., 2016; Taher et al., 2018a; Buttari et al., 2020). Patients with thalassemia are also at increased risk of silent brain infarcts. Several brain magnetic resonance imaging studies confirm a high prevalence of silent ischemic lesions in patients with thalassemia intermedia, especially in splenectomized adults who are non-transfusion-dependent and with elevated platelet counts (Taher et al., 2009; Musallam et al., 2012; Pazgal et al., 2016).

Iron overload and hemolysis may also contribute to the endothelial alterations and vascular inflammation, and iron chelation therapy has been found to improve rheological properties of blood, endothelial dysfunction and inflammatory processes in these syndromes (Taher et al., 2012; Saliba et al., 2015; Taher et al., 2018b; Marcon et al., 2018).

Since many abnormalities described in thalassemia major and intermedia may determine hemorheological alterations, in this study we have investigated hemorheological profiles of patients with thalassemia intermedia and thalassemia major to identify new indices of microvascular damage in thalassemia and explain the increased incidence of vascular complications. Furthermore, in order to evaluate the endothelial dysfunction or activation in thalassemia, levels of cytokines, growth factors, and adhesion molecules were investigated, together with the effects of therapies.

## Methods

This study included the analysis of blood samples from thalassemia patients and healthy individuals that were obtained according to guidelines established by the ethical committee for human subject studies, in accordance with the 1975 Helsinki Declaration, revised in 2008. Copies of informed consent can be provided on request. Thalassemia patients were monitored at the DH Thalassemia of S. Eugenio Hospital.

Diagnosis of thalassemia was made through a comprehensive assessment of clinical presentation and hematological and molecular characterization of globin mutations (Giardine, B et al., 2014; Greene et al., 2014; Risoluti R et al., 2016; Risoluti R et al., 2018). Clinical data included age, gender, splenectomy status, clinical complications, type of treatment received (RBC transfusion, iron chelation), C-reactive protein (CRP) values, serum ferritin levels, liver iron concentration (LIC) values. Clinical laboratory examinations also included levels of fibrinogen, C and S proteins, Antithrombin III, D Dimer, partial thromboplastin time (PTT) ratio, and international normalized ratio (INR).

### Hematological parameters and hemorheological profiles

A venous peripheral EDTA-anticoagulated blood sample was obtained from all patients and healthy subjects. The complete blood count was determined by ADVIA 120 (Siemens, United States).

The rheological profile was carried out by Rheo-Microscope (Anton Paar, Germany), that is, a glass parallel plates rheometer, Physica MCR301, with a Peltier system for temperature control (37°C ± 0.5°C). Whole blood viscosity (η) was determined at shear rates 1 s-1 (η1) and 200 s-1 (η200), low and high shear rates, respectively, according to the Recommendation of the International Committee for Standardization in Haematology (ICSH 1986) and the International Expert Panel for Standardization of Hemorheological Methods (Baskurt et al., 2009).

Blood viscosities η1 and η200 describe respectively the flow conditions of whole blood in the micro and macro circulation. Plasma viscosity (ηpl) testing was performed at shear rates 200 s-1. Erythrocyte Aggregation Index (EAI) was determined as η1 and η200 ratio (η1/η200). Since blood viscosity increases with the rise of hematocrit and thalassemia patients have hematocrit values around 30%, the hemorheological profile analysis was performed by determining blood viscosities and erythrocyte aggregation index in conditions of native and normalized (n) hematocrit (Hct) (e.g., adjusted to 40 or 45%, for females and males respectively).

The evaluation of RBCs viscoelastic properties was performed by analyzing elastic modulus G', and viscous modulus G", as a function of strain rate at a constant value of deformation amplitude, selected on the previously determined linear viscoelastic range by strain test. The frequency test was evaluated in the range from 0.1 to 10 Hz. (f = ω/2π). The values of the modules G' and G", expressed in Pa, were determined by oscillatory measurements in the range of viscoelastic linearity (10% deformation), as previously described (Caprari et al., 2019).

### Multiplex magnetic immunoassays

Concentration of cytokines (IL-2, IL-6, IL-10, IL-17A, and TNF- alpha), chemokines (IL-8, macrophage inflammatory protein (MIP)-1alpha), adipocytokines (leptin and adiponectin), growth factor (VEGF), and adhesion molecules (intercellular adhesion molecule (ICAM)-1 and VCAM-1) were determined in plasma obtained from 47 patients with thalassemia, and from 23 healthy subjects (CTR).

Commercially available multiplex magnetic immunoassay kits (Bio-Rad Laboratories, Hercules, CA) were used to quantify concentrations of human IL-2, IL-6, IL-8, IL-10, IL-17A, MIP-1alpha, TNF-alpha, VEGF (Bio-Plex Pro Human Cytokine 8-plex customized from 27-plex), leptin and adiponectin (Bio-Plex Pro Human Diabetes Assay Panel), ICAM-1 and VCAM-1 (Bio-PlexHuman Cytokine Group II 21-Plex Panel, ICAM-1 and VCAM-1).

The multi-analyte suspension array is based on Luminex’s XMAP Technology. The different analytes were quantified using the Bio-Plex protein array reader Bio-Plex^®^ 200 System (Bio-Rad) equipped with a magnetic washer, and analyzed by using a BioPlex Manager Software version 6.1.

Results were measured and expressed as the concentration of analytes (pg/ml). Values presenting a coefficient of variation beyond 8% were discarded before the final data analysis.

### Enzyme linked immunosorbent assays (ELISA)

Concentrations of the monocyte/macrophage activation marker CD163, the adhesion molecules E-selectin and L-selectin, and of the growth factor angiopoietin-1 were quantified in plasma obtained from the 47 patients and 21 healthy subjects by commercially available ELISA kits (R&D Systems, Inc. Minneapolis, MN, United States).

All samples were assayed in duplicate and all ELISA kits were performed according to manufacturer’s instructions.

### Statistical analysis

Data were analyzed using Student’s t-test or Mann-Whitney U test. The differences in the mean values of a given parameter between groups were evaluated applying the Student’s t-test for independent samples. For skewed distribution Kruskal–Wallis test was used. The correlations were determined using Pearson’s correlation coefficient, which was modified using Bonferroni’s multivariate adjustment. A *p*-value less than 0.05 was considered statistically significant. Statistical analyses were performed using GraphPad Prism four software (GraphPad Software Inc., La Jolla, CA, United States) and IBM SPSS v26 software.

## Results

### Characterization of thalassemia patients

Thalassemia group (47 patients with mean age of 39 ± 15 years with a male-to-female ratio of 19:28) included 18 thalassemia major (TM) patients regularly transfused (mean age of 35 ± 7 years), 18 patients with transfusion-dependent thalassemia intermedia (TDTI) (mean age of 44 ± 19 years), and 11 patients with non-transfusion-dependent thalassemia intermedia (NTDTI) (mean age of 35 ± 8 years). Three HbH thalassemia patients were included in the thalassemia intermedia groups, one patient was transfusion dependent, and two were non-transfusion dependent. Patients, disease, and treatment characteristics are shown in Table 1. The healthy individuals (n. 28) were blood donors with a mean age of 40 ± 11 years.

**TABLE 1:** Patients, disease, and treatment characteristics.

Characteristic	All patients (*n* = 47)	TM (*n* = 18)	TDTI (*n* = 18)	NTDTI (*n* = 11)
Age (mean ± SD)	39 ± 15	35 ± 7	44 ± 19	35 ± 8
Gender	28F/19M	9F/9M	11F/7M	11F/7M
Splenectomy, n (%)	35 (74.5)	16 (88.9)	12 (66.7)	7 (63.6)
Cholecystectomy, n (%)	18 (38.3)	5 (27.8)	11 ((61.1)	2 (18.2)
Hypogonadism, n (%)	13 (27.7)	12 (66.7)	1 (5.5)	—
Osteoporosis, n (%)	9 (19.1)	8 (44.4)	1 (5.5)	—
TEE, n (%)	6 (12.8)	2 (11.1)	3 (16.7)	1 (9.1)
Hypothyroidism, n (%)	5 (10.6)	5 (27.8)	—	—
Diabetes, n (%)	5 (10.6)	3 (16.7)	2 (11.1)	—
CRP mean median (mg/dl) range	0.35	0.34	0.37	0.27
	0.14	0.09	0.24	0.09
	0.00–3.08	0.00–3.08	0.02–1.41	0.09–0.72
Serum Ferritin mean median (ng/ml) range	662	318	937	512
	436	400	486	277
	67–4438	67–790	120–4438	160–2159
LIC, mg Fe/g dw	2.34 ± 1.97	1.57 ± 1.19*	2.94 ± 2.36*	2.70 ± 2.08
Iron chelator, n (%)	41 (87.2)	18 (100)	18 (100)	5 (45.5)
DFO	11 (23.4)	7 (38.9)	4 (22.2)	—
DFX	18 (38.3)	4 (22.2)	10 (55.5)	4 (36.4)
DFP	2 (4.2)	2 (11.1)	—	—
DFO + DFX	4 (23.5)	1 (5.5)	2 (11.1)	1 (9.1)
DFO + DFP	6 (12.8)	4 (22.2)	2 (11.1)	—

TM, Thalassemia major; TDTI, Transfusion-dependent thalassemia intermedia; NTDTI, Non-transfusion-dependent thalassemia intermedia. SD, Standard deviation; TEE, Thromboembolic event; CRP, C-reactive protein; DFO, Deferoxamine; DFX, Deferasirox; DFP, Deferiprone. *p < 0.05, Student’s t-test.

A total of 35 (74.5%) patients were splenectomized (TM, *n* = 16; TDTI, *n* = 12; NTDTI, *n* = 7). The most common disease-related complications were cholelithiasis with cholecystectomy in 18 (38%) patients, mainly in TDTI patients (61%).

Hypogonadism, osteoporosis, and hypothyroidism were observed in 13 (27.7%), 9 (19.1%), and 5 (10.6%) patients, respectively (mainly TM), diabetes was observed only in 5 (10.6%) patients (TM, *n* = 3; TDTI, *n* = 2), and TEE in 6 (12.8%) patients (TM, *n* = 2; TDTI, *n* = 3; NTDTI, *n* = 1).

High levels of serum ferritin were found in TDTI (mean 937 ng/ml, range 120–4438 ng/ml) and TM (mean 512 ng/ml, range 160–2159 ng/ml) patients. Mean LIC values were higher in TDTI and NTDTI patients than in TM patients and a significant difference was observed (*p* < 0.05) between TDTI and TM patients.

Regular transfusions were received by the TM and TDTI patients, and only occasional transfusions for transient severe anemia secondary to infections, pregnancy or surgery were received by the NTDTI patients, who were studied far from transfusion.

C-reactive protein mean values were comparable between the different patients’ groups and a low grade inflammation was evident. The iron chelation therapy is summarized in Table 1. Forty-one patients (87%) received iron chelators, and the most used chelators were deferasirox (DFX) and deferoxamine (DFO) that were administered to 18 and 11 patients respectively, only in few cases combined iron chelator therapy was used (Table 1).

All the coagulation parameters were within the reference intervals except for the C protein which had decreased in the thalassemia groups.

### Hematological parameters

**FIGURE 1:**
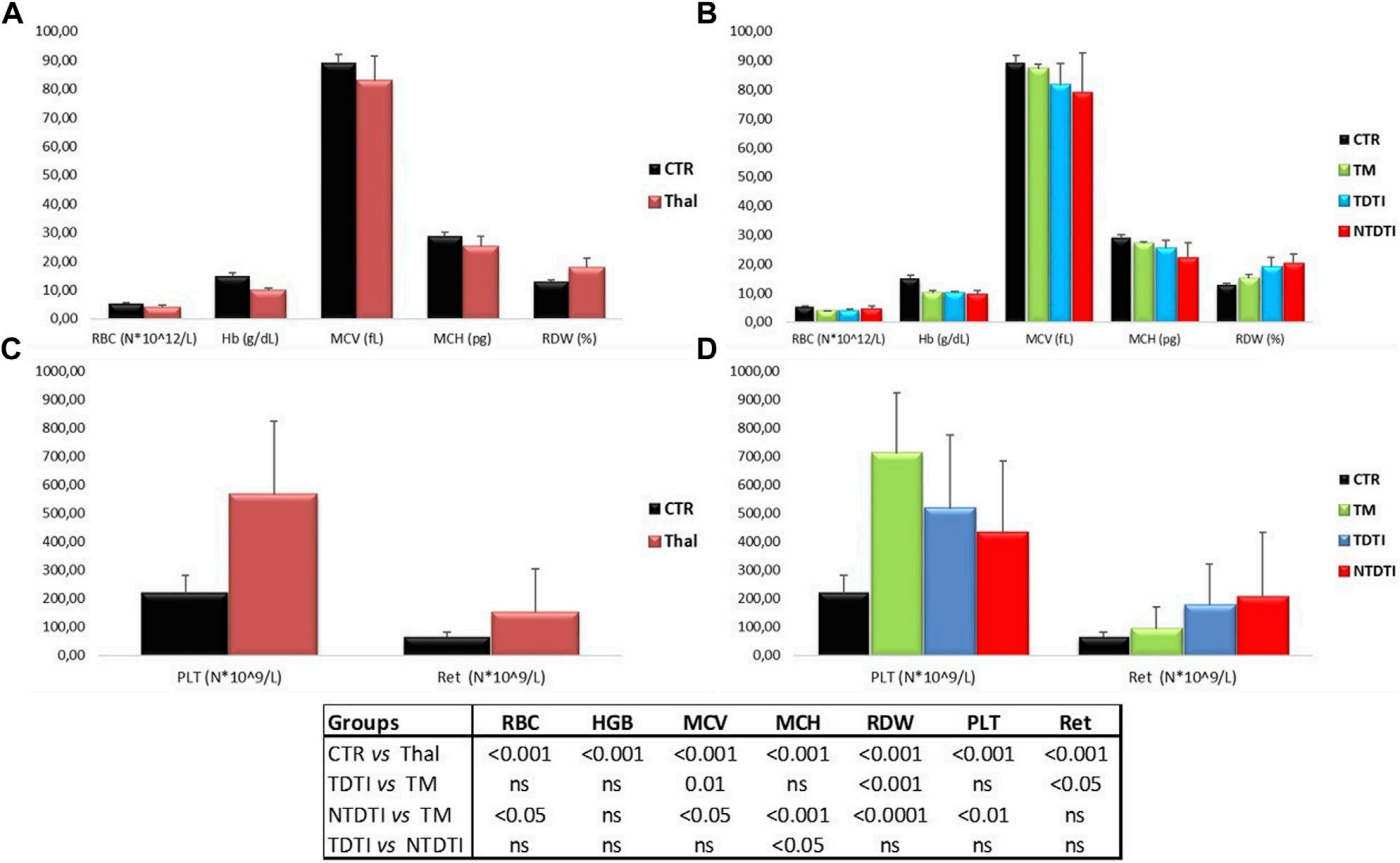
Hematological parameters from thalassemia patients and healthy subjects: **(A)** and **(C)** Comparisons between healthy subjects (CTR) and all thalassemia patients (Thal); **(B)** and **(D)** Comparisons between CTR and patients groups divided according to the severity of the disease: TM: thalassemia major; TDTI: transfusion-dependent thalassemia intermedia; NTDTI: non-transfusion-dependent thalassemia intermedia; RBC: red blood cell; Hb: Hemoglobin; MCV: mean cell volume; MCH:mean cell hemoglobin; RDW: Red blood cells Distribution Width; PLT: platelet count; Ret: reticulocyte count; ns: not significant.

Highly significant differences in hematological parameters have been observed between healthy subjects (CTR) and all thalassemia patients (Thal) (Figures 1A,C).

The comparison between the thalassemia groups (TM, TDTI, NTDTI) is shown in Figures 1B,D. Levels of hemoglobin were comparable among the thalassemia groups. Significant differences for the MCV, RDW and reticulocyte counts were evident between TDTI and TM. Both TM and TDTI groups of patients had high platelet counts, but the difference between them was not significant. Highly significant differences in hematological parameters were observed between TM and NTDTI patients. NTDTI group demonstrated significant increases in RBC, RDW and reticulocyte counts, and significant lower values in MCV, MCH and PLT values compared to the TM group. Comparable hematological parameters were observed between NTDTI and TDTI patients with only a significant lower value of MCH in the NTDTI patients (Figures 1B,D).

### Hemorheological profiles

**FIGURE 2:**
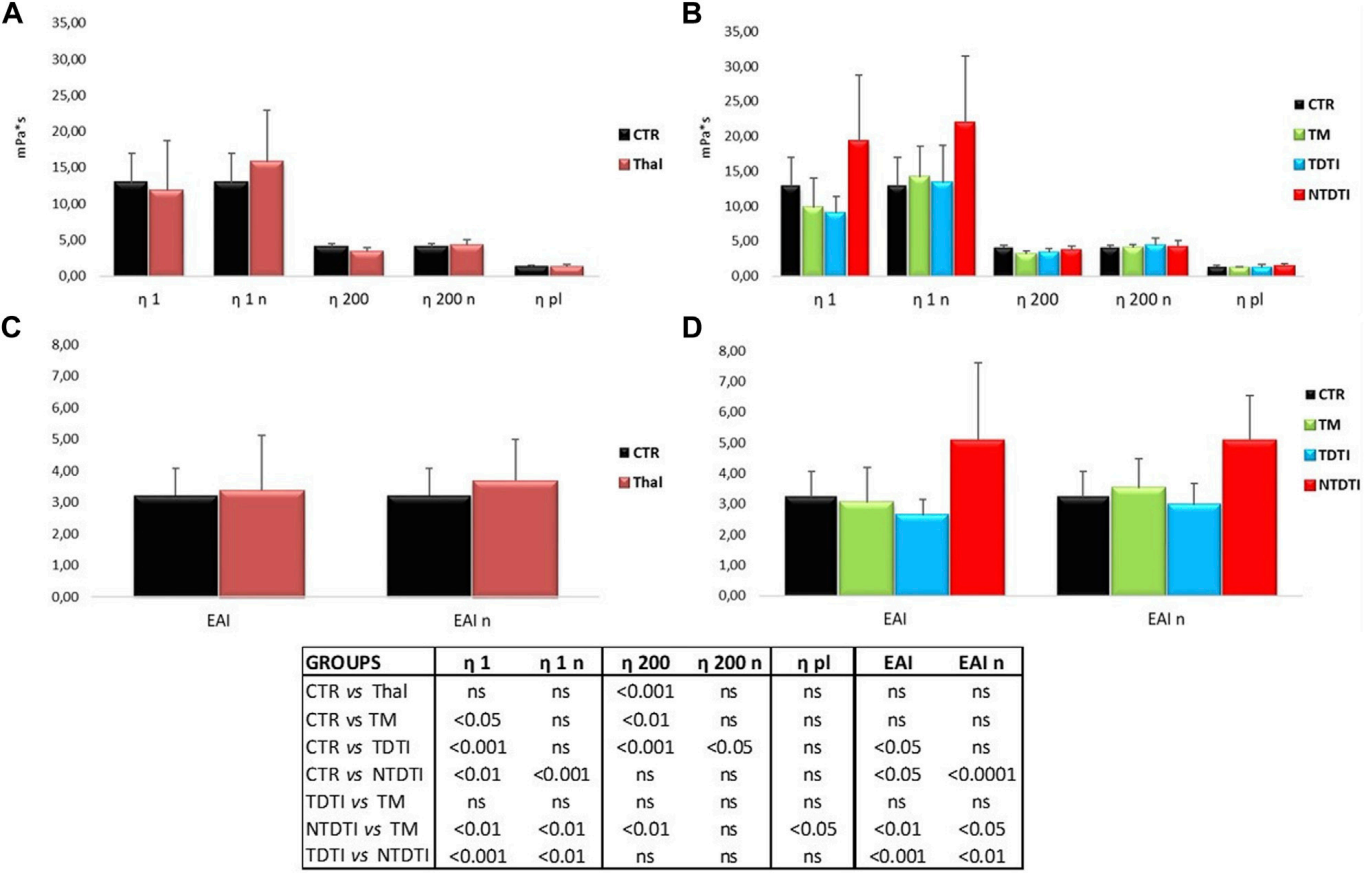
Blood viscosities η1 and η200, obtained at low (1 s-1) and high (200 s-1) shear rates, and the erythrocyte aggregation index (EAI) from thalassemia patients and healthy subjects determined at native and normalized (n) hematocrit. **(A)** and **(C)** Comparisons between healthy subjects (CTR) and all thalassemia patients (Thal). **(B)** and **(D)** Comparisons between CTR and patients groups divided according to the severity of the disease: TM: thalassemia major; TDTI: transfusion-dependent thalassemia intermedia; NTDTI: non-transfusion-dependent thalassemia intermedia; ns: not significant; η1 and η1n: native and normalized hematocrit blood viscosities at low shear rates; η200 and η200n: native and normalized hematocrit blood viscosities at high shear rates; EAI and EAIn determinaed at native and normalized hematocrit.

Figures 2A–D show the blood viscosities η1 and η200, obtained at low (1 s-1) and high (200 s-1) shear rates, and the erythrocyte aggregation index (EAI) of thalassemia patients and healthy subjects determined at native and normalized (n) hematocrit. The parameters have been compared between healthy subjects and all thalassemia patients (Figures 2A,C), and between the different thalassemia groups (Figures 2B,D).

The blood viscosity η200 of the thalassemia patients determined in condition of native hematocrit was lower than that of the controls, but the comparison at normalized hematocrit showed a marked increase in viscosity at low shear rates η1 in thalassemia, even if the large variability of the data in the patients.

When the same parameters were compared in patients divided in different groups, many significant differences were evident between healthy subjects and thalassemia patients and between the different types of thalassemia. The most significant differences were observed in the NTDTI group: the η1 value was much higher than the value of healthy subjects, and significantly different from both native and normalized hematocrit compared to the values determined in healthy, TM and TDTI patients. This increase in η1 resulted in a corresponding significant increase in EAI in NTDTI patients. The values of η200 of NTDTI patients were comparable with those of healthy subjects and TDTI patients and significantly higher than TM patients.

There were no differences in rheological parameters between TDTI and TM patients, while the differences between NTDTI and TM patients were generally significant for all parameters (η1, η200, EAI and also plasma viscosity). The comparison between NTDTI and TDTI patients showed highly significant differences for η1 and EAI.

It should be noted that the two NTD α-thalassemia patients (HbH) had very high η1 and EAI values, while the other regularly transfused HbH patient had a rheological profile comparable to those of healthy subjects.

**FIGURE 3:**
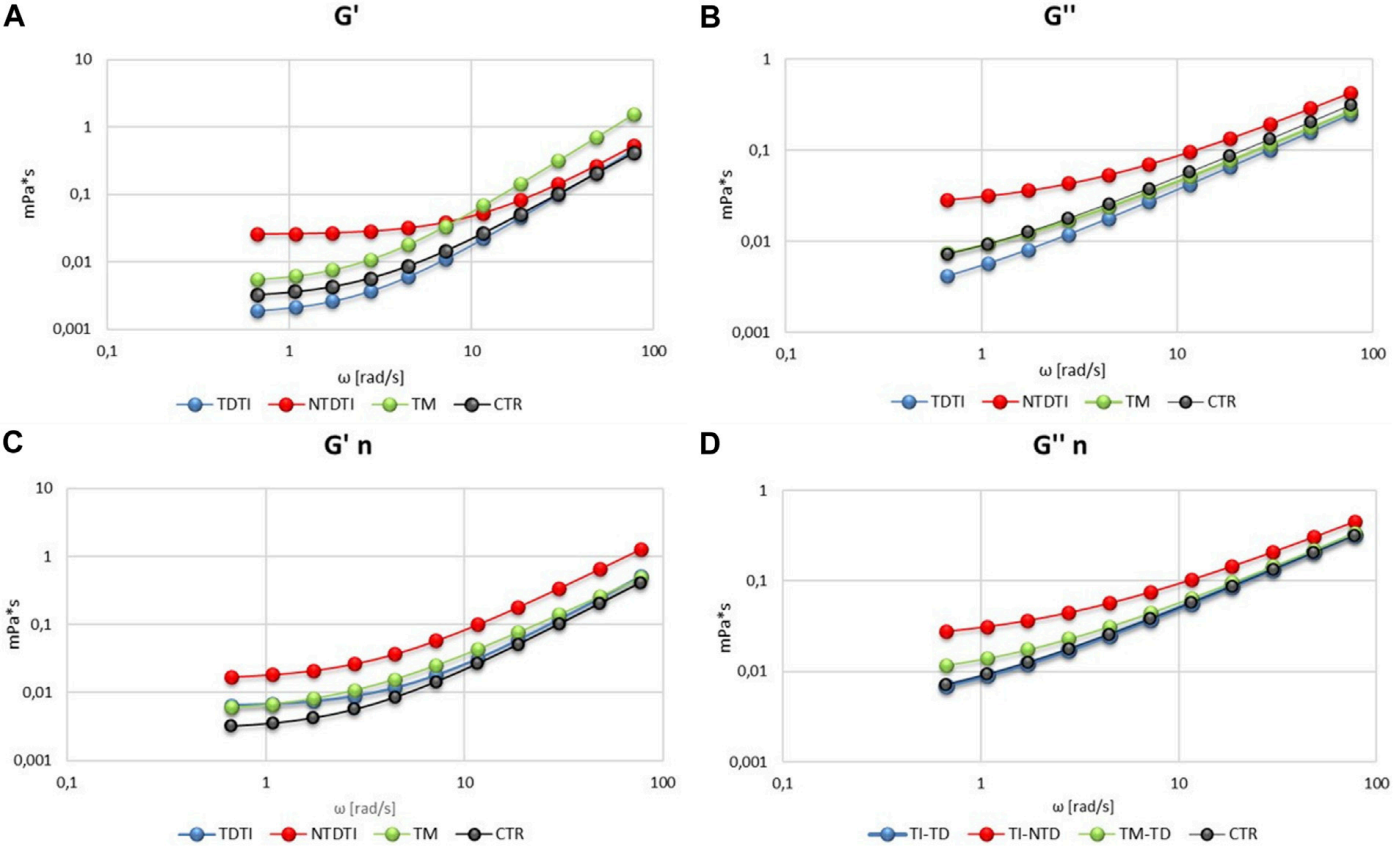
Viscoelastic profiles (storage modulus G' and loss modulus G'') of blood from thalassemia groups and healthy subjects at native **(A,B)** and normalized (n) hematocrit **(C,D)**. TM: thalassemia major; TDTI: transfusion-dependent thalassemia intermedia; NTDTI: non-transfusion-dependent thalassemia intermedia.

The viscoelastic properties have been determined as elastic modulus G' and viscous modulus G'' and the Figure 3 shows the mean curves of the values obtained from the different groups analyzed. It is possible to note the difference in the values of the G' and G" between the controls and the different thalassemia groups at native and normalized hematocrit.

The elastic modulus G′ (Figure 3A) shows that transfusion-dependent thalassemia patients (both TDTI and TM) have a viscoelastic profile comparable to that of CTR, but with a high modulus in TM at high ω values. On the other hand, G' profile of NTDTI patients is quite different, showing a marked slip limit, and higher values of G′ at low ω in comparison with CTR, TDTI and TM groups, and values of G' comparable to those of CTR and TDTI at high ω values (Figure 3A). These differences are also noted in the viscous modulus G′ (Figure 3B), where the profile of NTDTI falls in a much higher range, denoting an increase in erythrocyte viscosity throughout the profile.

The mean curves of G′ and G′ obtained at normalized hematocrit (Figures 3C,D) do not show particular differences in deformability and viscosity between CTR and TDTI and TM, while confirm the higher values of the elastic and viscous modules in the NTDTI group.

### Iron chelation therapy and hemorheological parameters

**FIGURE 4:**
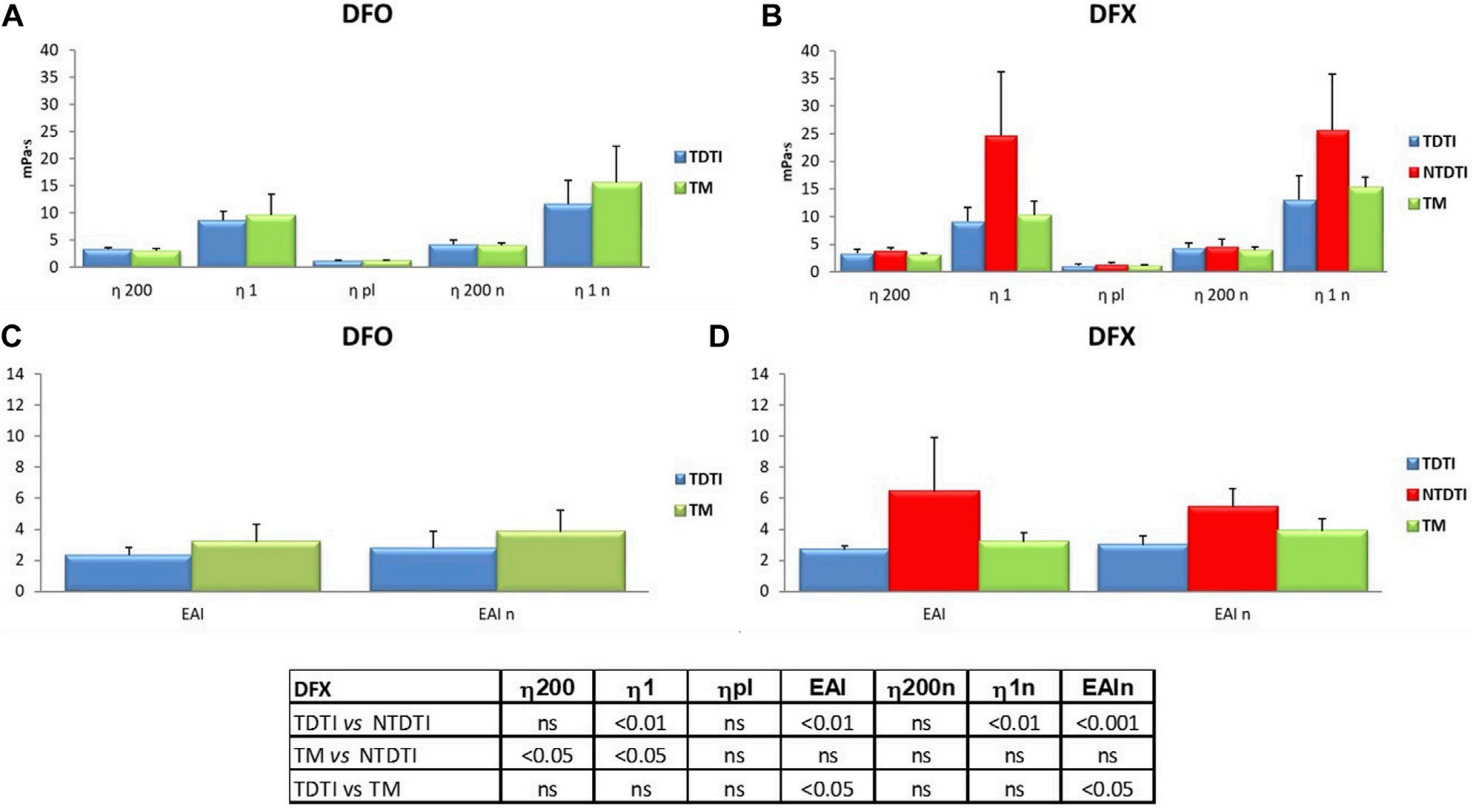
The effect of iron chelation therapy on the hemorheological profiles of patients with thalassemia intermedia and major. Deferasirox (DFX) and deferoxamine (DFO) were administered as mono therapy to 18 and 11 patients, respectively; η1 and η1n: native and normalized hematocrit blood viscosities at low shear rates; η200 and η200n: native and normalized hematocrit blood viscosities at high shear rates; EAI and EAIn erythrocyte aggregation index determinaed at native and normalized hematocrit. The comparison between the thalassemia groups showed significant statistical differences in the patients using DFX; TM: thalassemia major; TDTI: transfusion-dependent thalassemia intermedia; NTDTI: non-transfusion-dependent thalassemia intermedia; ns: not significant.

The effect of iron chelation therapy on the hemorheological profiles of patients with thalassemia intermedia and major was investigated (Figure 4). Forty-one patients received iron chelators, and the most used drugs were deferasirox (DFX) and deferoxamine (DFO) that were administered as mono therapy to 18 and 11 patients respectively, while only in few patients combined iron chelator therapy was used (Table 1).

There are no significant differences in viscosity parameters between all the thalassemia patients treated with different therapies as confirmed by the statistical analysis (data not shown), while the comparison between the different thalassemia groups showed statistically significant differences in the patients using DFX.

In Figure 4 the rheological parameters from thalassemia patients treated with DFO and DFX are compared. The mean η1 value of patients treated with DFX (Figure 4B) is higher than the values from DFO treated patients (Figure 4A), depending on the presence of NTDTI patients in the DFX group, since DFX is used to treat excess free iron in some cases of particularly hemolytic NTDTI patients.

In the DFX group we have found significant differences between TDTI and NTDTI patients with regard to η1 and erythrocyte aggregation index at native and normalized hematocrit (*p* < 0.01) (Figures 4B,D), and between TM and NTDTI for η200 and η1 (*p* < 0.05) (Figure 4B). Moreover an increase in EAI was observed in TM patients as compared with TDTI patients (*p* < 0.05) (Figure 4D).

### Levels of soluble markers

Circulating levels of cytokines, chemokines and adhesion molecules in plasma from patients and healthy subjects are reported in Tables 2, 3; Supplementary Figure S1. Analysis of cytokines showed a wide variability of the IL-6 and IL-10 values both in the group of total thalassemia patients (THAL) and in healthy subjects (CTR), with extremely high values of IL-6 in some patients. However the concentrations of the pro-inflammatory cytokine IL-6 resulted significantly lower in THAL than in CTR (*p* < 0.05) (Table 2; Supplementary Figure S1).

**TABLE 2:** Circulating levels of cytokines, chemokines and adhesion molecules in plasma from patients and healthy subjects. Data are reported as medians and range values.

Circulating marker	CTR *n* = 23	THAL *n* = 47	TM *n* = 18	TDTI *n* = 18	NTDTI *n* = 11
IL-6 (pg/ml)	17.81 (0.05–639)	4.64 (0.05–2511)	7.26 (0.05–470)	1.24 (0.05–56.61)	4.64 (0.05–2511)
IL-10 (pg/ml)	0.04 (0.01–3.93)	0.01 (0.01–4.06)	0.06 (0.01–1.69)	0.01 (0.01–0.37)	0.37 (0.01–4.06)
IL-8 (pg/ml)	0.04 (0.04–28.28)	0.04 (0.04–19)	0.65 (0.04–3.57)	0.04 (0.04–19)	0.04 (0.04–10.74)
VCAM-1 (pg/ml)	298065 (181944–437814)	392869 (209257–629912)	365009 (209257–583678)	377158 (268055–629912)	453553 (312135–609001)
ICAM-1 (pg/ml)	214921 (56608–302961)	250730 (111456–479618)	229262 (111456–479618)	259290 (200977–407296)	264367 (170298–407700)

CTR, Healthy subjects; THAL, Total thalassemia patients; TM, Thalassemia major; TDTI, Transfusion dependent thalassemia intermedia; NTDTI, Non-transfusion dependent thalassemia intermedia.

**TABLE 3:** Circulating levels of adhesion molecules, growth factors, adipocytokines and the macrophage activation marker in plasma from patients and healthy subjects. Data are reported as medians and range values.

Circulating marker	CTR *n* = 21	THAL *n* = 47	TM *n* = 18	TDTI *n* = 18	NTDTI *n* = 11
E-selectin (ng/ml)	30 (14–67.9)	45.2 (22.1–84.8)	42.85 (22.2–69.6)	53.35 (22.1–84.8)	32.9 (23.20–70.80)
L-selectin (ng/ml)	517 (388–768)	668 (296–1462)	621 (296–1036)	843 (376–1462)	523 (320.3–979)
VEGF (pg/ml)	10.7 (1.52–36.36)	35.95 (4.06–151.8)	30.68 (6.98–151.8)	51.13 (4.06–126.2)	22.97 (4.06–146.5)
Ang-1 (pg/ml)	2810 (544–9000)	7294 (188–9000)	8855 (1799–9000)	6871 (339–9000)	2083 (1898–8878)
Leptin (pg/ml)	1256 (185–10410)	395 (1.8–9526)	322 (1.8–3615)	331 (13.9–9526)	872 (12.01–2378)
CD163 (ng/ml)	508 (380–997)	808 (272–1435)	685 (272–1346)	918 (448–1435)	620 (428–890)

CTR, Healthy subjects; THAL, Total thalassemia patients; TM, Thalassemia major; TDTI, Transfusion dependent thalassemia intermedia; NTDTI, Non-transfusion dependent thalassemia intermedia; Ang-1, Angiopoietin-1.

As concern the chemokines and the adipocytokines, we observed that IL-8 and leptin, similarly to IL-6, resulted significantly lower in total patients than in CTR (IL-8, *p* < 0.05; leptin *p* < 0.01) (Tables 2, 3; Supplementary Figure S1) Conversely, levels of all the analyzed growth factors, adhesion molecules and of CD163 resulted higher in patients than in CTR (VEGF, *p* < 0.001; ang-1, *p* < 0.05; VCAM-1, *p* < 0.001; ICAM-1, *p* < 0.05; E-selectin, *p* < 0.01; L-selectin, *p* < 0.05) (Tables 2, 3; Supplementary Figure S1).

To evaluate if soluble marker levels were associated to the clinical severity of the disease, we compared the different groups of patients (TM, TDTI, and NTDTI) and the results are reported in Tables 2, 3; Supplementary Figure S2. We observed that IL-6 and IL-10 concentrations were significantly lower in TDTI patients than in CTR (IL-6, *p* < 0.05; IL-10, *p* < 0.05) (Table 2; Supplementary Figure S2), and the highest values of IL-6 observed in the THAL patients (Supplementary Figure S1) belonged to NTDTI group. The concentration of the anti-inflammatory cytokine IL-10 in patients with NTDTI and TM were significantly higher than in the TDTI patients (*vs*. TM, *p* < 0.05; *vs*. NTDTI, *p* < 0.001) (Supplementary Figure S2).

Furthermore, analysis in patient groups revealed that the lower levels of IL-8 and leptin observed in total patients compared to healthy subjects revealed that these differences were only due to patients with TM (IL-8, *p* < 0.05; leptin, *p* < 0.001) (Tables 2, 3; Supplementary Figure S2).

Analysis of growth factors, adhesion molecules and of CD163 circulating levels showed that VEGF, VCAM-1 and CD163 were significantly higher in all the groups of patients (VEGF: TM *p* < 0.01, TDTI *p* < 0.0001, NTDTI *p* < 0.05; VCAM-1: TM *p* < 0.01, TDTI *p* < 0.001, NTDTI *p* < 0.0001; CD163: TM *p* < 0.01, TDTI *p* < 0.0001, NTDTI *p* < 0.05) (Tables 2, 3; Supplementary Figure S2).

Ang-1 and E-selectin were higher only in patients with TM and TDTI (ang-1: TM *p* < 0.001, TDTI *p* < 0.05; E-selctin TM *p* < 0.01, TDTI *p* < 0.001), whereas ICAM-1 and L-selectin only in patients with TDTI (ICAM-1 *p* < 0.01; L-selectin *p* < 0.001) (Tables 2, 3; Supplementary Figure S2).

Of note, levels of L-selectin and CD163 in patients with TDTI were significantly higher than those observed in the other two groups of patients for L-selectin (*vs*. TM, *p* < 0.011; *vs*. NTDTI, *p* < 0.05), and in patients with NTDTI for CD163 (*p* < 0.01) (Supplementary Figure S2). Circulating levels of the biomarkers IL-2, IL-17A, TNF-alpha, MIP-1alpha and adiponectin did not significantly differ among groups (data not shown).

Analysis of correlations between circulating marker pairs determined in plasma samples obtained from patients (Supplementary Table S1) showed significant positive correlations between the cytokines IL-6 and IL-10 (*p* < 0.001). These markers negatively correlated with the growth factor ang-1 (IL-6 *vs*. ang-1, *p* < 0.05), the adhesion molecule L-selectin (IL-10 *vs*. L-selectin, *p* < 0.05), and with the monocyte/macrophage activating factor CD163 (IL-10 *vs*. CD163, *p* < 0.05). Also, the chemokine IL-8 negatively correlated with ang-1 (*p* = 0.05).

Positive correlations were observed also between the adhesion molecules VCAM-1 and ICAM-1 (*p* < 0.001), between E-selectin and ang-1 (*p* < 0.01), and between L-selectin and CD163 (*p* < 0.01). The same analysis in samples from healthy subjects showed again a positive correlation between the cytokines IL-6 and IL-10 (*p* < 0.01), and between the adhesion molecules VCAM-1 and ICAM-1 (*p* < 0.05). Furthermore, in healthy subjects population, the adhesion molecules E-selectin and L-selectin positively correlated each other (*p* < 0.05), and negatively correlated with the adhesion molecules ICAM-1 and VCAM-1, and with the growth factor VEGF (E-selectin *vs*. ICAM-1 *p* < 0.01; L-selectin *vs*. VCAM-1 *p* < 0.05; E-selectin *vs*. VEGF *p* = 0.05) (Supplementary Table S1).

### Analysis of soluble marker levels in patients divided according to therapy

When we compared soluble marker levels in patients divided according to the presence or not of splenectomy, we observed significantly higher levels of ang-1 in splenectomized patients than in non-splenectomized ones (*p* < 0.0001, Table 4; Supplementary Figure S3A). In patients divided according to the severity of the disease, this difference resulted statistically significant only in the group with TDTI (*p* < 0.0001, Supplementary Figure S3A).

**TABLE 4:** Circulating angiopoietin-1 and VEGF levels in plasma from healthy subjects and from patients divided according to the occurrence/absence of splenectomy and to therapy with aspirin, respectively. Data are reported as median, and range values.

Circulating marker	Treatment	CTR	THAL	TM	TDTI	NTDTI
Ang-1 (pg/ml)	Splenectomy	*n* = 0	*n* = 36	*n* = 17	*n* = 12	*n* = 7
		-	7869 (860–9000)	9000 (1799–9000)	8143 (5107–9000)	3960 (860–8878)
	No splenectomy	*n* = 20	*n* = 11	*n* = 1	*n* = 6	*n* = 4
		2810 (544–9000)	1099 (189–4391)	4391	1215 (339–3744)	874.1 (189–3715)
VEGF (pg/ml)	ASA therapy	*n* = 0	*n* = 26	*n* = 11	*n* = 10	*n* = 5
		-	29.87 (4.1–80.0)	25.41 (13.2–45.7)	51.13 (4.1–80.0)	20.93 (4.1–37.6)
	No ASA therapy	*n* = 20	*n* = 21	*n* = 7	*n* = 8	*n* = 6
		10.7 (1.52–36.36)	38.38 (7.0–152)	39.19 (7.0–152)	54.76 (11.5–126.2)	35.14 (9.5–146.5)

CTR, Healthy subjects; THAL, Total thalassemia patients; TM, Thalassemia major; TDTI, Transfusion dependent thalassemia intermedia; NTDTI, Non-transfusion dependent thalassemia intermedia; Ang-1, Angiopoietin-1; ASA, Aspirin.

Analysis of data in accordance to pharmacological treatments showed that patients taking aspirin had significant decreased levels of the growth factor VEGF in comparison to patients who did not take it (*p* < 0.05, Table 4; Figure 3B). No differences were observed when patients were divided according to the type of iron chelation therapy (data not shown).

### Analysis of correlations between soluble markers, hemorheological parameters, ferritin levels, and LIC values

Analysis of correlations between circulating markers and hemorheological parameters are shown in Table 5. Significant positive correlations between η1 or η1/η200 and the cytokines IL-6 and IL-10 were observed in healthy subjects (η1*vs*. IL-6 and *vs*.IL-10, *p* < 0.001; η1/η200 *vs*. IL-6, *p* < 0.01; η1/η200 *vs*. IL-10, *p* < 0.05). In thalassemia patients η1and η1/η200 correlated positively with the cytokines IL-6 and IL-10 (*p* < 0.001), and negatively with the adhesion molecule L-selectin (η1, *p* < 0.05; η1/η200, *p* < 0.05).

**TABLE 5:** Pearson’s correlation coefficients and *p* values between circulating markers, hemorheological parameters, ferritin levels, and LIC values in patients and healthy subjects.

Marker	THAL										CTR					
	η1		η1/η200		η pl		Ferritin		LIC		η1		η1/η200		η pl	
IL-6	0.581	<0.001	0.650	<0.001	0.062	NS	−0.096	NS	0.280	NS	0.982	<0.001	0.910	<0.01	0.674	NS
IL-10	0.615	<0.001	0.683	<0.001	0.051	NS	−0.034	NS	0.230	NS	0.971	<0.001	0.876	<0.05	0.667	NS
IL-8	0.022	NS	0.046	NS	0.594	<0.001	0.096	NS	−0.108	NS	0.362	NS	0.153	NS	0.027	NS
L-selectin	0.354	<0.05	0.332	<0.05	0.149	NS	0.298	NS	0.178	NS	0.628	NS	0.808	NS	0.202	NS
Ang-1	−0.140	NS	−0.183	NS	−0.172	NS	−0.232	NS	−0.398	<0.05	0.228	NS	−0.032	NS	0.897	NS
CD163	−0.271	NS	−0.251	NS	−0.213	NS	0.344	<0.05	0.167	NS	−0.331	NS	−0.564	NS	0.518	NS

THAL, Total thalassemia patients; CTR, Healthy subjects; LIC, Liver iron concentration; Ang-1, angiopoietin-1; NS, Not significant.

In this regard, it should be noted that in the NTDTI group with the highest IL-6 values (Supplementary Figure S2) we have also observed high blood viscosity values of η1 and erythrocyte aggregation index (η1/η200). Furthermore, the plasma viscosity (ηpl) positively correlated with the chemokine IL-8 (*p* < 0.001).

In thalassemia patients we also observed a significant positive correlation between ferritin levels and CD163 concentrations (*p* < 0.05) and a negative correlation between LIC values and ang-1 (*p* < 0.05) (Table 5). In healthy subjects ferritin levels and LIC values were not evaluated.

## Discussion

### Hemorheological alterations in thalassemia

The rheological properties of blood play an important role in regulating blood flow in micro and macro circulation. Since blood viscosity is an important determinant of flow resistance, an increase in blood viscosity can adversely affect and worsen circulatory failure (Baskurt et al., 2007; Cokelet et al., 2007). Some studies have shown that in thalassemia syndromes red blood cells exhibit altered hemodynamic properties that facilitate microcirculatory diseases: increased aggregation and reduced deformability, as well as a marked increase in adherence to the vascular endothelial cells (Pérez et al., 2004; Pérez et al., 2006; Baskurt et al., 2007; Cooke et al., 2007).

Iron overload and hemolysis also contribute to endothelial alterations and vascular inflammatory state (Cappellini et al., 2010; Cappellini et al., 2012; Taher et al., 2018a). Both transfusions and therapeutic approaches, such as splenectomy, seem to influence the onset of vascular complications such as thrombosis, heart attacks, thromboembolic events due to a state of hypercoagulability. Regularly transfused thalassemia patients have a decrease in the incidence of thromboembolic phenomena compared to non-transfusion dependent patients, and patients with thalassemia intermedia seem to have an increased predisposition to thrombosis compared to patients with thalassemia major (Taher et al., 2006; Cappellini et al., 2010; Cappellini et al., 2012; Taher et al., 2012; Taher et al., 2015). Moreover it has also been observed that iron chelation therapy can improve the rheological properties of blood, endothelial dysfunction and inflammatory processes in thalassemia syndromes (Musallam et al., 2012; Saliba et al., 2015; Taher et al., 2015).

In this study the hemorheological profiles of thalassemia patients have been characterized in order to point out new indices of vascular impairment in thalassemia and to compare the effectiveness of the different iron chelating therapies in relation to rheological parameters. For this purpose we have compared healthy subjects with both all thalassemia patients and thalassemia patients divided according to the severity of disease, i.e., thalassemia major, transfusion dependent thalassemia intermedia, and non-transfusion-dependent thalassemia intermedia.

The differences in the hematological pictures between thalassemia major and thalassemia intermedia have been confirmed, the two groups of patients are distinguished from each other by the erythrocyte indices, percentages of reticulocytes and platelets counts. In particular, the more consistent increase in RDW and reticulocytes in TI patients highlights the presence of marked hemolysis, while the higher values of MCV in TM patients are essentially linked to the effect of constant transfusion therapy. High values of platelets are characteristic in splenectomized patients.

The three groups of thalassemia have different hemorheological profiles that do not depend only on the type of molecular mutation, as transfusion therapy, the intake of different iron chelators, splenectomy, and comorbidities can have a relevant role.

The analysis of blood viscosities shows an increase in viscosity when we compare the viscosities obtained at the native hematocrit (about 30%), characteristic of thalassemia, and normalized one (40%–45%). While transfusion-dependent thalassemia patients, both TM and TI, have blood viscosities comparable to those of healthy subjects, NTDTI patients are significantly different from other regularly transfused TI patients showing much higher viscosity values at low shear rates (η1), corresponding to the flow conditions of the microcirculation, and increase in erythrocyte aggregation, and plasma viscosity.

The analysis of the viscoelastic characteristics, which allows to release the elastic component G′ (which gives information on the erythrocyte deformability) from the viscous component G′, confirms different behaviors of the thalassemia groups. In the TM group, the G' curve is increased compared to the native hematocrit of the controls, indicating an increase in the elastic modulus, an index of greater stiffness of red blood cells. In the TDTI group, the increase in G' is observed at normalized hematocrit and is already evident at low ω, but comparable with the TM group. Much more evident is the behavior of NTDTI erythrocytes which have both very high elastic and viscous modules. The shift of the G' and G'' modules to progressively higher values is consistent with the increase in blood viscosity, which we observed under different shear conditions in the normalized hematocrit.

In conditions of low deformation rate, the blood viscosity is mainly conditioned by the aggregation of the cells, therefore the small increase is presumably related to an increase in the adhesion forces between the erythrocytes. This greater tendency to aggregation and the reduced deformability of the red blood cells suggest the consequences of these anomalies. Circulating erythrocytes could form larger and more irregular aggregates, held together by greater cohesive forces which could result in a slowdown or even blockage of blood flow, especially in microcirculation, with consequent reduction of tissue perfusion. The morphological and rheological modifications could, therefore, alter the fluid dynamic characteristics of the erythrocytes with negative repercussions such as, for example, thromboembolic events.

The results of this study indicate that patients with non-transfusion dependent thalassemia intermedia present important blood rheological changes which may explain the higher frequency of thromboembolic events observed in the literature. Hemorheological analyzes are therefore an extremely useful means of evaluating the fluid dynamic characteristics in thalassemia syndromes and to prevent the onset of such events.

As regards iron chelating therapies, generally personalized and tailored to the specific clinical and hematological problems for each patient, our results, which concern a small number of patients by type of therapy, did not show significant differences in rheological behavior and the effectiveness of the different iron chelating therapies in relation to rheological parameters seems to be the same. The higher values observed in the group treated with DFX are due to the presence in this group of NTD patients, who appear to have a more altered hemorheological profile.

### Behavior of inflammation markers in thalassemia

In this study we have also evaluated the levels of a panel of circulating biomarkers in thalassemia patients with the aim to establish a relationship between the levels of these biomarkers and severity of the disease. In these patients, infections, immune system abnormalities, hypercoagulable state and an increased risk of thrombosis are considered the main causes of morbidity and mortality, where infectious complications could be the results of functional alteration in the immune system (Moshtaghi-Kashanian et al., 2006; Cappellini et al., 2012).

The circulating biomarkers studied are the main components of the immune and endothelial systems or are related to vascular inflammation, and play fundamental roles in the pathogenesis of inflammatory disorders. The results of our study show that patients with thalassemia have a systemic alteration of some circulating biomarkers in comparison to healthy blood donors. The panel of circulating biomarkers considered includes cytokines and chemokines which are mainly produced by immune cells and are involved in several important biological activities such as the recruitment of immune and hematological cells to the injured tissues. Among these soluble immune mediators, we found statistically significant differences for the cytokines IL-6 and IL-10 and for the chemokine IL-8 whose levels resulted decreased in patients with respect to healthy subjects. In particular, we found that the decrease in these molecules levels was statistically significant only in the group of patients with transfusion-dependent thalassemia intermedia (IL-6 and IL-10) and in the group of patients with thalassemia major (IL-8), thus indicating that multi-transfusions could be responsible for changes in the subsets of immune cells responsible for their productions. IL-6 and IL-8 are important components of the inflammatory response whereas IL-10 is an immune regulatory cytokine that modulates immunity towards an anti-inflammatory response. Results of reduced IL-10 levels in patients and particularly in patients with TDTI may in part explain the presence of increased inflammation and a more severe disease reported in these patients. The decrease in the pro-inflammatory molecules IL-6 and IL-8, observed in TD patients’ groups needs to be further investigated. We can hypothesize that the reduction of all these mediators is the result of the toxic effect on immune cells of iron overload caused by blood transfusions and ineffective erythropoiesis leading to increased iron absorption from gut. Although therapy with iron chelators can contrast excessive iron overload, thalassemia patients are characterized by increased iron concentrations in many organs, particularly liver, heart and pancreas (Gülhan et al., 2016).

Our results of reduced IL-6, IL-10, and IL-8 serum levels in beta thalassemia disagree with findings of previous studies in this field, which are themselves controversial, probably due to differences in patient populations and therapies. Findings in poly-transfused patients with thalassemia major demonstrated that serum levels of IL-6 are undetectable or within the normal range (Lombardi et al., 1994), whereas other results showed an increase in the circulating levels of this cytokine in the same category of patients (Oztürk et al., 2001; Aggeli et al., 2005; Moshtaghi-Kashanian et al., 2006; El-Rasheidy et al., 2016). Contrasting results are also reported for the anti-inflammatory and immunoregulatory cytokine IL-10, able to decrease immune responses. IL-10 was found to be increased in thalassemia patients with a possible role in the inhibition of erythropoiesis and in the induction of anemic crisis in these patients (Butthep et al., 2015). In contrast, a more recent study reported undetectable serum levels of IL-10 in patients with TM (Gülhan et al., 2016). No differences between patients and controls in serum levels of this cytokine were reported by other studies (Del Vecchio et al., 2002; Moshtaghi-Kashanian et al., 2006; Balouchi et al., 2014). As concern IL-8, previous studies reported undetectable levels of IL-8 in the 77% of the patient population (Gülhan et al., 2016) or increased plasma levels of this chemokine in patients with thalassemia intermedia and major as compared to healthy subjects (Uguccioni et al., 1993; Dore et al., 1995; Oztürk et al., 2001).

The adipocytokines leptin and adiponectin, produced by adipose cells, represent a link between metabolism, immunity and chronic inflammation (Chaliasos et al., 2010). In our study leptin resulted decreased in patients with the more severe form of thalassemia (TM) with respect to healthy subjects, indicating the impact of genotype and poly-transfusions in this alteration. Leptin has an important role in the regulation of appetite, body fat mass, and endocrine function and suppresses inflammation in the heart muscle, thus protecting heart from diseases.

Our results agree with literature data on significantly lower serum leptin in patients when compared to controls (Karachaliou et al., 2006; Chaliasos et al., 2010; Shahramian et al., 2013; Elsayh et al., 2016). The low leptin in thalassaemia has been ascribed to the toxic effect of iron overload on adipose tissue (Chaliasoset al. 2010). In contrast to studies reporting higher levels of adiponectin in patients (Chaliasos et al., 2010; El-Rasheidy et al., 2016; Elsayh et al., 2016), we did not find any significant differences among patient groups and controls.

Thalassemia syndromes are characterized by high degree of endothelial activation and damage, vascular inflammation and hypercoagulability, which explain the high incidence of thromboembolic events, such as stroke, deep venous thrombosis and pulmonary hypertension, mainly in patients with thalassemia intermedia (Cappellini et al., 2009). There is evidence that thalassemia intermedia is associated with disturbances in vascular endothelial cell proliferation (Shitrit et al., 2008). Endothelial activation is reflected by increased serum levels of soluble endothelial adhesion molecules such as ICAM-1 and VCAM-1, E-selectin and L-selectin. Another important mediator is VEGF that plays several roles in angiogenesis (Fahmey et al., 2013). VEGF levels have been related to the clinical severity of thalassemia intermedia, as expressed by the degree of hepatomegaly and splenectomy and cardiac indexes (Shitrit et al., 2008). There are findings demonstrating that another pro-angiogenic factor, angiopoietin-1 displaying potent pro-angiogenic activity, and significantly increasing endothelial cell proliferation, migration and capillary-like structure formation, is present in elevated concentrations in patients with steady-state sickle cell anemia (Duits et al., 2006; Lopes et al., 2015). In our study we have observed a negative correlation between Ang-1 and LIC values in thalassemia patients, Also hemolysis and free plasma hemoglobin are implicated in the vasculopathy associated to sickle cell disease and beta-thalassemia. CD163, the main hemoglobin-haptoglobin receptor expressed on monocytes/macrophages, plays an important role in the clearance of hemoglobin and its determination may be useful to better understand hemolysis-related endothelial dysfunction. Of note, high levels of soluble CD163 are a marker of coronary atherosclerosis (Durda et al., 2022) and we have found in thalassemia patients CD163 concentrations significantly correlated with ferritin levels. This result is in accordance with previous studies demonstrating the association between CD163+ macrophages and ferritin in human atherosclerotic plaques (Li and Yuqan, 2005). This soluble molecule can be considered also a biomarker for pulmonary hypertention and vaso-occlusive complications in patients with sickle cell disease (Tantawy et al., 2012). Our data showed that VCAM-1, VEGF and CD163 are elevated in all patient groups, thus representing markers associated to thalassemia, independently from severity of the disease. In contrast, the increased levels of E-selectin and angiopoietin-1 observed in TM and TDTI patients, but not in NTDTI ones, when compared to healthy subjects indicate a possible association of these molecules to the presence of repetitive transfusions. Of note, the data on increased levels of ICAM-1 and L-selectin associated to low concentrations of IL-10 and IL-6 only in TDTI patients indicate that particularly these patients have an altered systemic vascular inflammation. This concept is sustained by the higher levels of L-selectin and CD163, and lower levels of IL-10 observed in TDTI when compared to NTDTI.

Therapeutic approaches like splenectomy and transfusions seem to influence the manifestation of thromboembolic complications (Kanavaki et al., 2009). Splenectomized patients present higher risks for thrombosis than non-splenectomized patients, for the presence of increased numbers of damaged erythrocytes and thrombocyte counts. In our study, splenectomized patients with TDTI showed increased levels of the pro-angiogenic factor angiopoietin 1. As angiopoietin is produced also by megacariocytes and platelets (Li JJ et al., 2001), the increased number of these elements in splenectomized patients may explain our results. We also observed lower levels of L-selectin in all splenectomized patients with respect to not splenectomized ones.

Of note we observed lower levels of VEGF in patients under aspirin treatment, suggesting the possible role of this drug in contributing to control endothelial activation and angiogenesis in patients with beta-thalassemia. This result is in line with previous observations demonstrating that aspirin treatment significantly decreased plasma levels of VEGF in patients with myocardial ischemia or with hypertension (Gerraha et al., 2004; Nadar et al., 2006). VEGF is a heparin-binding glycoprotein secreted by different types of cells, particularly vascular smooth muscle cells, megakaryocytes, lymphocytes, macrophages and neutrophils. As it has been demonstrated that platelet activation has a main role in determining circulating VEGF level increase (Kranz-Rau C et al., 2000), the association between reduced VEGF levels and aspirin treatment has been ascribed to the antiplatelet effect of this drug.

In conclusion, this study has demonstrated that patients with transfusion-dependent and non-transfusion dependent thalassemia intermedia may present important hemorheological changes and these alterations, that have an important role in the circulation, are correlated with the endothelium activation as demonstrated by the circulating inflammation markers studied. Our results may explain the reason of the increased frequency of thromboembolic events in thalassemia intermedia previously observed. The evaluation of the hemorheological profiles in thalassemia patients can provide useful new indicators of vascular impairment and disease severity in thalassemia in order to prevent the onset of such events.

## Data Availability

The original contributions presented in the study are included in the article/Supplementary Material, further inquiries can be directed to the corresponding author.
