# Supplementary material for: Hemorheological profiles and chronic inflammation markers in transfusion-dependent and non-transfusion- dependent thalassemia
Source: Front Mol Biosci. 2023 Jan 9;9:1108896. doi: 10.3389/fmolb.2022.1108896 (PMC9868635; doi:10.3389/fmolb.2022.1108896)
Supplement: Supplementary file 1 [file DataSheet1.docx]

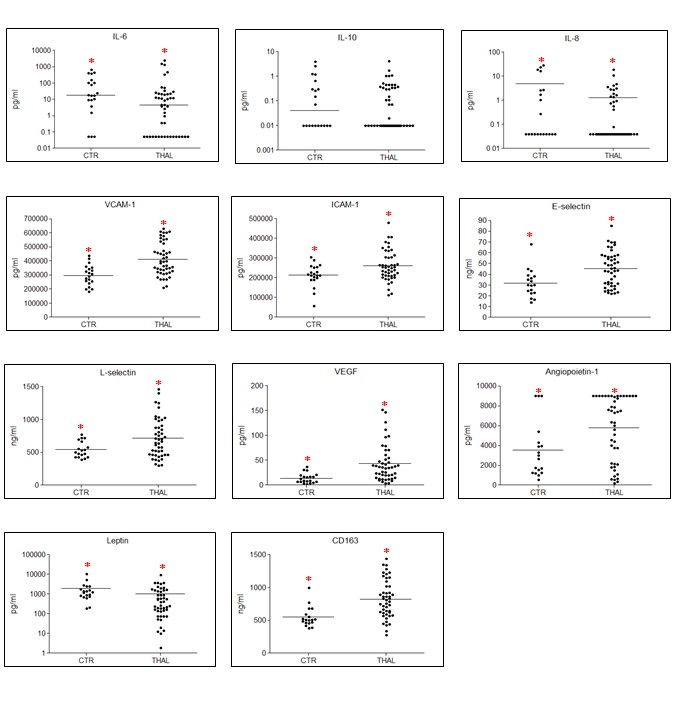


**Figure S1:** Circulating cytokine levels in plasma from patients and healthy subjects. CTR: healthy subjects; THAL: all thalassemia patients. * p<0.05.


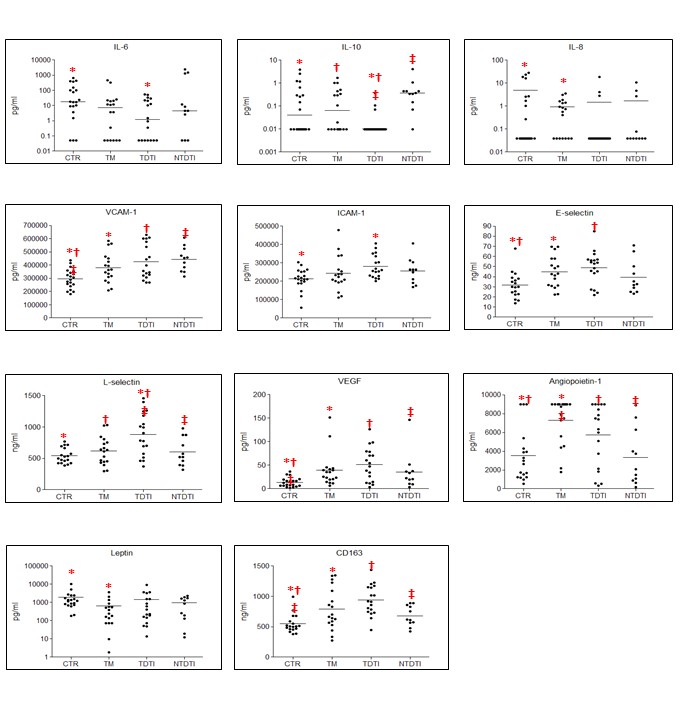


**Figure S2:** Circulating cytokine levels in plasma from patients divided according to the severity of the disease and from healthy subjects. CTR: healthy subjects; TM: thalassemia major; TDTI: transfusion-dependent thalassemia intermedia; NTDTI: non-transfusion-dependent thalassemia intermedia. *, †, ‡ P<0.05.


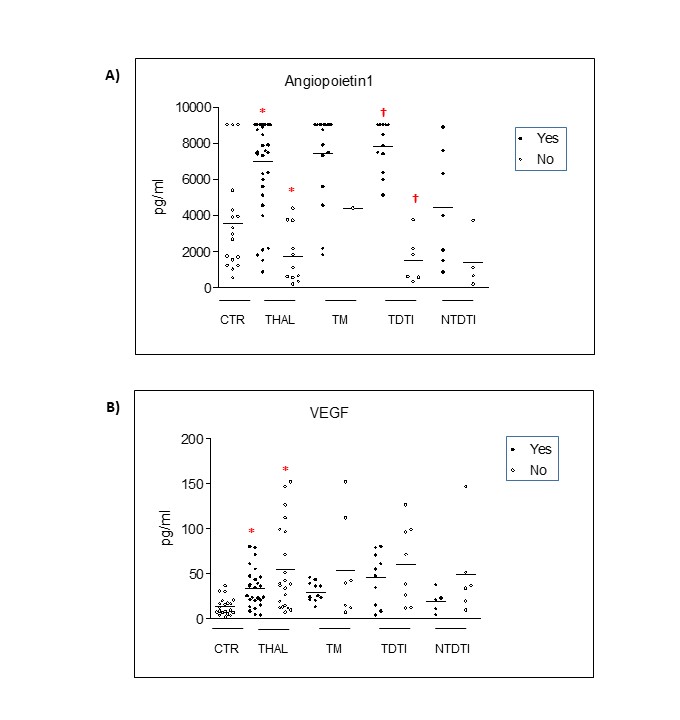


**Figure S3:** Circulating angiopoietin-1 and VEGF levels in plasma from patients divided according to the therapy. A) Angiopoietin-1 levels in patients divided according to the occurrence/absence of splenectomy, and in healthy subjects. B) VEGF levels in patients divided according to therapy with aspirin, and in healthy subjects. CTR: healthy subjects; TM: thalassemia major; TDTI: transfusion-dependent thalassemia intermedia; NTDTI: non-transfusion-dependent thalassemia intermedia; *, † P<0.05

**Table S1:** Pearson’s correlation coefficients and p values between circulating marker pairs determined in plasma samples obtained from patients and healthy subjects. THAL: total thalassemia patients; CTR: healthy subjects; NS: not significant.

| **Correlations** | **THAL** | | **CTR** | |
| --- | --- | --- | --- | --- |
|  | **R** | **P** | **R** | **P** |
| IL-6 *vs* IL-10 | 0.785 | **<0.001** | 0.596 | **<0.01** |
| ICAM-1 *vs* VCAM-1 | 0.681 | **<0.001** | 0.515 | **<0.05** |
| E-selectin *vs* ICAM-1 | 0.080 | NS | -0.893 | **<0.01** |
| L-selectin *vs* E-selectin | 0.137 | NS | 0.541 | **<0.05** |
| L-selectin *vs* VCAM-1 | -0.165 | NS | -0.804 | **<0.05** |
| L-selectin *vs* IL-10 | -0.303 | **<0.05** | -0.109 | NS |
| VEGF *vs* E-selectin | -0.073 | NS | -0.707 | **<0.05** |
| Angiopoietin1 *vs* E-selectin | 0.388 | **<0.01** | -0.050 | NS |
| Angiopoietin1 *vs* IL-6 | -0.326 | **<0.05** | 0.070 | NS |
| Angiopoietin1 *vs* IL-8 | -0.287 | **<0.05** | 0.094 | NS |
| CD163 *vs* L-selectin | 0.477 | **<0.01** | -0.030 | NS |
| CD163 *vs* IL-10 | -0.313 | **<0.05** | -0.075 | NS |
